# Use of nonsteroidal anti-inflammatory drugs and risk of oral cancer: a cohort study

**DOI:** 10.1038/sj.bjc.6603250

**Published:** 2006-07-25

**Authors:** S Friis, A Poulsen, L Pedersen, J A Baron, H T Sørensen

**Affiliations:** 1 Institute of Cancer Epidemiology, Department of Genetics and Medicine, Danish Cancer Society, DK-2100 Copenhagen, Denmark; 2 Department of Clinical Epidemiology, Aarhus University Hospital, Ole Worms Allé 1150, DK-8000 Aarhus C, Denmark; 3 Departments of Medicine and Community and Family Medicine, Dartmouth Medical School, Dartmouth College, Hanover 03755-3861, NH, USA

**Keywords:** nonsteroidal anti-inflammatory drugs, oral cancer, population based, risk, cohort study

## Abstract

Epidemiologic data regarding the chemopreventive potential of nonsteroidal anti-inflammatory drugs (NSAIDs) against oral cancer are sparse. We found a relative risk for oral cancer of 1.2 (95% CI, 1.0–1.6) among 169 589 Danish NSAID users (≥2 prescriptions), with no apparent trends in subgroups. Our study provided no clear evidence that NSAIDs may protect against oral cancer.

There is substantial experimental and epidemiological evidence that aspirin and other nonsteroidal anti-inflammatory drugs (NSAIDs) protect against colorectal adenomas and cancer (Baron, 2003; Ulrich *et al*, 2006). Epidemiologic studies have also consistently pointed to inverse associations between NSAID use and cancers of the stomach and oesophagus, whereas the results are mixed for other cancer sites (Gonzalez-Perez *et al*, 2003). Studies of animal models and human cancer cell lines have indicated that the potential chemopreventive effect of NSAIDs might extend to oral cancer (Goodin and Shiff, 2004; Wang, 2005), and this year a large phase III prevention trial of COX-2 inhibitors in patients with premalignant oral lesions (leukoplakia) is scheduled to be launched (Nelson, 2006). Interestingly, only a few epidemiological studies have provided data on the relationship between NSAID use and oral cancer, and the data are conflicting (Thun *et al*, 1993; Bosetti *et al*, 2003; Sørensen *et al*, 2003). This paucity of epidemiologic data prompted us to examine the incidence of oral cancer in a large cohort of NSAID users in Denmark.

## MATERIALS AND METHODS

The study was carried out within the population of North Jutland County, Denmark (population approximately 490 000 inhabitants), during the study period 1 January 1991 to 31 December 2002. From the files of the Danish Civil Registration System, established in 1968 (Frank, 2000), we identified all individuals in the county who were 16 years or older during the study period and resident in the county on 1 January 1991. We then excluded all individuals with a history of cancer (except nonmelanoma skin cancer) before study entry (1991 or age 16 years) by linkage to the Danish Cancer Registry, which has had accurate and almost complete nationwide ascertainment of cancer cases since 1943 (Storm *et al*, 1997). The final study population comprised 442 654 individuals.

The Danish National Health Service provides tax-supported health care for all inhabitants and refunds part of patient expenditures on a wide range of prescribed drugs, including non-aspirin NSAIDs. In Denmark, non-aspirin NSAID can be obtained only by prescription, except for low doses of ibuprofen which account for approximately 14% of the total use (Sørensen *et al*, 2003). All health-related services are registered to individual patients by use of a civil registry number assigned to all Danish citizens, which encodes gender and date of birth. In North Jutland County, pharmacy data are transferred to a research prescription database, which was initiated in 1989 and covered all pharmacies by 1991 (Gaist *et al*, 1997). The database holds key information on all prescriptions for refundable drugs dispensed from pharmacies in the county, including the type of drug prescribed according to the ATC classification system (World Health Organisation, 2001), date of dispensing at the pharmacy, and the civil registry number. Use of the civil registry number allows for the establishment of complete prescription histories for each individual and secures valid linkage between population-based registers.

Using the files of the prescription database and the cancer registry, we identified 169 589 individuals in the study population who had redeemed two or more non-aspirin NSAID prescriptions (ATC codes, M01A) during the study period, and who were free of cancer at the date of their second prescription. For all individuals in the study population, we also obtained information on diagnoses of chronic obstructive pulmonary disease (COPD) from the County Hospital Discharge Registry, a computerised database containing information on all non-psychiatric hospital admissions (since 1977) and outpatient visits (since 1995) in the county (Skriver *et al*, 2005).

All individuals in the study population were followed from 1 January 1991 or age 16 years, whichever occurred latest, until date of primary oral (mouth and tongue) cancer, other primary cancer (except nonmelanoma skin cancer), death, migration, or 31 December 2002, whichever occurred first. The person time of the study subjects was distributed according to use of NSAIDs in exposed time (≥2 prescriptions) (NSAID use) and unexposed time (no prescriptions) (nonuse). Person time between first and second NSAID prescription was excluded from the analyses to reduce the possible association between NSAIDs and oral cancer due to confounding by indication.

We calculated age- and gender-standardised incidence rates of oral cancer by applying direct standardisation in gender-specific 5-year age groups to the age distribution in the entire study population. Age- and gender-standardised incidence rate ratios (IRR) were computed by dividing incidence rates of oral cancer for NSAID users with incidence rates for nonusers. We categorised NSAID use into two groups based on the number of redeemed prescriptions (2–9 prescriptions, 10+ prescriptions). We further performed a subanalysis according to a history of COPD (diagnosis in the Hospital Discharge Registry during the period 1977–2002), as an indirect control for smoking.

## RESULTS

We identified 75 cases of oral cancer among NSAID users during 977 627 person-years of follow-up (mean, 5.8 years) and 110 cases among nonusers during 2 521 145 years of follow-up. The age-and gender-standardised incidence rate was 6.1 per 100 000 person-years among NSAID users and 4.9 per 100 000 person-years among nonusers. The table presents the relative risk estimates for oral cancer according to use of NSAIDs (Table 1). All IRR estimates were close to one and stratification by number of prescriptions or history of COPD revealed no apparent trends, although the IRR estimates for COPD patients tended to be slightly lower than those for the full cohort. The results did not change materially when stratified according to gender (data not shown).

## DISCUSSION

Our large population-based study based on registers with essentially complete and unbiased ascertainment of prescriptions, hospitalisations and outpatient visits for COPD, and cancer outcomes provided no evidence that NSAID intake prevents oral cancer. The incidence of oral cancer is increasing in many countries, and the lack of efficient therapy and the considerable associated morbidity and mortality make chemoprevention attractive (Goodin and Shiff, 2004; Wang, 2005), particularly since this might be targeted to high-risk individuals with leukoplakia or other precursor lesions (Nelson, 2006). NSAIDs have shown promise as chemopreventive agents for oral cancer in experimental studies (Goodin and Shiff, 2004), but the available epidemiologic data including the present analysis do not support a major protective effect of NSAIDs against oral cancer.

Our findings may have been influenced by differences in NSAID use according to important risk factors for oral cancer, notably smoking and alcohol consumption. We were only able to indirectly control for smoking by examining risk in a subset of patients hospitalised with COPD, of whom the large majority can be assumed to have been smokers. Our inability to adjust for actual use of tobacco, alcohol consumption, and over-the-counter purchase of low-dose ibuprofen and aspirin may have obscured a preventive effect of NSAID use against oral cancer. However, analyses of US health care utilisation data sets indicate that these potential confounders have limited influence in studies of NSAID use and various health outcomes (Schneeweiss *et al*, 2005). Also, our finding of no substantial trends in IRRs over number of NSAID prescriptions argues against a major protective effect of NSAIDs on the development of oral cancer. The lack of any clear effect even after stratification by history of COPD similarly suggests no appreciable confounding by smoking, although these analyses were based on small numbers. Further epidemiological studies with large sample sizes and comprehensive information on potential confounders may be warranted to evaluate the chemopreventive potential of NSAIDs against oral cancer.

## Figures and Tables

**Table 1:** Age- and gender-standardised rate ratios for oral cancer among NSAID users in North Jutland County, Denmark, 1991–2002

	**No. of cases**	**Person-years**	**Standardised rate ratio (95% CI)**
All			
No prescriptions	110	2 521 145	Reference
2+ prescriptions	75	977 627	1.2 (1.0–1.6)
2–9 prescriptions	17	192 241	1.0 (0.6–1.6)
10+ prescriptions	58	785 386	1.3 (1.0–1.6)
			
*No chronic obstructive pulmonary disease* *			
No prescriptions	102	2 436 925	Reference
2+ prescriptions	66	919 129	1.2 (0.9–1.5)
2–9 prescriptions	13	174 921	0.9 (0.5–1.6)
10+ prescriptions	53	744 208	1.3 (1.0–1.7)
			
*Chronic obstructive pulmonary disease*			
No prescriptions	8	84 220	Reference
2+ prescriptions	9	58 499	0.9 (0.4–1.7)
2–9 prescriptions	4	17 320	1.2 (0.3–3.1)
10+ prescriptions	5	41 179	0.8 (0.2–1.8)

* No hospitalisation for chronic obstructive pulmonary disease.
