# Use of nonsteroidal anti-inflammatory drugs and risk of oral cancer: a cohort study

**DOI:** 10.1038/sj.bjc.6603488

**Published:** 2006-11-28

**Authors:** S Friis, A Poulsen, L Pedersen, J A Baron, H T Sørensen

**Correction to**: *British Journal of Cancer* (2006) **95**, 363–365. doi:10.1038/sj.bjc.6603250

Owing to an author error, there was a mistake in the presentation of results in Table 1 in the above paper. All figures in the table were correct, but the number of cases, person-years and rate ratios presented for NSAID users with 2–9 prescriptions were those pertaining to NSAID users with 10+ prescriptions, and vice versa. Correction of this interchange of results has no implications for the presentation of results in the article text, and conclusions and abstract remain the same. The correct Table 1 is shown below.

## Figures and Tables

**Table 1:** Age- and gender-standardized rate ratios for oral cancer among NSAID users in North Jutland County, Denmark, 1991–2002

	**No. of cases**	**Person-years**	**Standardised rate ratio (95% CI)**
*All*			
No prescriptions	110	2 521 145	Reference
2+ prescriptions	75	977 627	1.2 (1.0–1.6)
2–9 prescriptions	58	785 386	1.3 (1.0–1.6)
10+ prescriptions	17	192 241	1.0 (0.6–1.6)
			
*No chronic obstructive pulmonary disease* a			
No prescriptions	102	2 436 925	Reference
2+ prescriptions	66	919 129	1.2 (0.9–1.5)
2–9 prescriptions	53	744 208	1.3 (1.0–1.7)
10+ prescriptions	13	174 921	0.9 (0.5–1.6)
			
*Chronic obstructive pulmonary disease*			
No prescriptions	8	84 220	Reference
2+ prescriptions	9	58 499	0.9 (0.4–1.7)
2–9 prescriptions	5	41 179	0.8 (0.2–1.8)
10+ prescriptions	4	17 320	1.2 (0.3–3.1)

a No hospitalisation for chronic obstructive pulmonary disease.

